# Microbial niche differentiation explains nitrite oxidation in marine oxygen minimum zones

**DOI:** 10.1038/s41396-020-00852-3

**Published:** 2021-01-06

**Authors:** Xin Sun, Claudia Frey, Emilio Garcia-Robledo, Amal Jayakumar, Bess B. Ward

**Affiliations:** 1 Department of Geosciences, Princeton University, Princeton, NJ 08544 USA; 2 Department of Biology, Calle Republica Saharaui, University of Cadiz, 3.11519 Puerto Real, Cadiz Spain; 3 Present Address: Department of Environmental Science, University of Basel, Basel, Switzerland

**Keywords:** Biogeochemistry, Water microbiology, Microbial ecology

## Abstract

Nitrite is a pivotal component of the marine nitrogen cycle. The fate of nitrite determines the loss or retention of fixed nitrogen, an essential nutrient for all organisms. Loss occurs via anaerobic nitrite reduction to gases during denitrification and anammox, while retention occurs via nitrite oxidation to nitrate. Nitrite oxidation is usually represented in biogeochemical models by one kinetic parameter and one oxygen threshold, below which nitrite oxidation is set to zero. Here we find that the responses of nitrite oxidation to nitrite and oxygen concentrations vary along a redox gradient in a Pacific Ocean oxygen minimum zone, indicating niche differentiation of nitrite-oxidizing assemblages. Notably, we observe the full inhibition of nitrite oxidation by oxygen addition and nitrite oxidation coupled with nitrogen loss in the absence of oxygen consumption in samples collected from anoxic waters. Nitrite-oxidizing bacteria, including novel clades with high relative abundance in anoxic depths, were also detected in the same samples. Mechanisms corresponding to niche differentiation of nitrite-oxidizing bacteria across the redox gradient are considered. Implementing these mechanisms in biogeochemical models has a significant effect on the estimated fixed nitrogen budget.

## Introduction

Nitrogen is required for all life and its availability in natural systems affects global climate through control of biological carbon cycling and nitrous oxide fluxes. The loss and retention of fixed inorganic nitrogen are thought to be spatially separated: loss is restricted to anoxic environments, while the oxidation of nitrite (NO_2_^−^) to nitrate (NO_3_^−^) by nitrite-oxidizing bacteria (NOB) is considered to be an aerobic process. Anaerobic NO_2_^−^ oxidation in anoxic layers of marine oxygen minimum zones (OMZs) has been suggested [1–3], but has not been experimentally proven. It could not be shown that NO_2_^−^ oxidation is independent of O_2_ because O_2_ concentrations were not measured in the experimental samples. Unavoidable O_2_ contamination below previous analytical detection limits during sampling cannot be quantified but may be enough to support measured NO_2_^−^ oxidation rates. NO_2_^−^ oxidation by microaerophilic NOB has been recognized [4, 5] and may explain some apparently anaerobic NO_2_^−^ oxidation rates in oxic–anoxic interfaces of OMZs. Whether measured NO_2_^−^ oxidation rates in anoxic seawaters were also fueled by O_2_ introduced by sampling and incubation manipulations or were actually anaerobic rates remains unknown. Contradictory results in O_2_ manipulation experiments complicate the story: additions of O_2_ to samples from oxic–anoxic interfaces caused decreased [3, 6] or increased [5] NO_2_^−^ oxidation, suggesting that these dynamic environments contain diverse NOB with different O_2_ preferences.

Diverse NOB were recently detected in both oxic [7] and anoxic seawater [8] via single-cell sequencing and metagenomics. Among the seven genera of NOB, *Nitrococcus* and *Nitrospina*-like NOB or *Candidatus* Nitromaritima were found in hypoxic and anoxic seawater [6, 8–10]. Dominant OMZ NOB were absent in oxic oceanic regions, and NOB from oxic seawater were rare in OMZs [8]. The difference in NOB communities under different conditions suggests niche differentiation among marine NOB. It also implies that different NOB may have different O_2_ sensitivities and substrate affinities.

To explore whether the composition of the NOB assemblage could be responsible for the previous contradictory results, we investigated substrate kinetics and effects of O_2_ on NO_2_^−^ oxidation at depths with distinct redox features. We sampled the oxycline (oxic), the top of the anoxic oxygen deficient zone (ODZ, the oxic–anoxic interface) and the anoxic ODZ core at two stations in the Eastern Tropical North Pacific (ETNP) OMZ to capture different responses by the diverse NOB communities occupying distinct niches. ODZ, also known as anoxic marine zones (AMZs) [11], refers to the anoxic zone of the OMZ and OMZ refers to an oceanic region that includes an ODZ and oxic water layers. In addition, to determine whether NO_2_^−^ oxidation could occur without O_2_, we measured NO_2_^−^ oxidation rates in ^15^N stable isotope tracer experiments with samples collected from anoxic ODZs while monitoring O_2_ using highly sensitive LUMOS sensors (detection limit ≈1 nM) [12, 13].

## Results and discussion

### Substrate kinetics and the effects of O_2_ addition on NO_2_^−^ oxidation

**Fig. 1:**
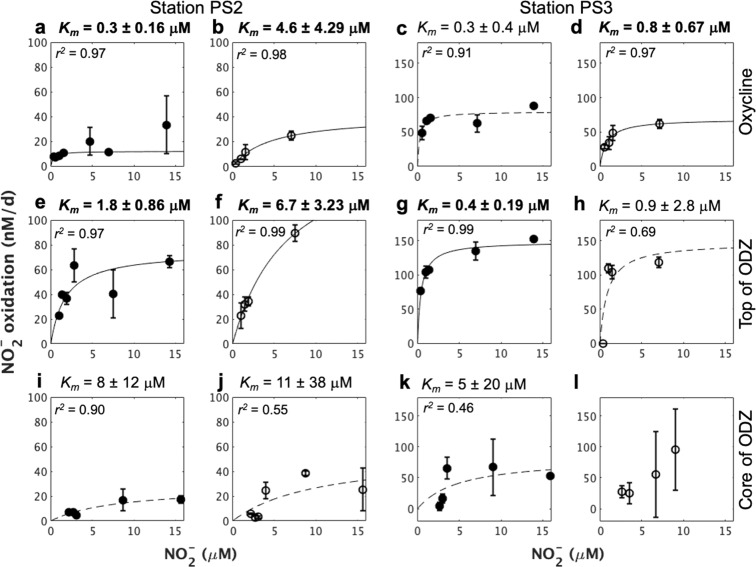
Nitrite kinetics of NO_2_^−^ oxidation rates at stations PS2 and PS3. Samples were from oxycline (**a**, **b**, **c**, **d**), ODZ top (**e**, **f**, **g**, **h**), and ODZ core (**i**, **j**, **k**, **l**), respectively. Closed circles indicate incubation under low O_2_ conditions (0.3–0.7 μM), and open circles indicate high O_2_ conditions (2.3–8.7 μM). Solid lines indicate a *K*_*m*_ significantly larger than zero. The adjusted R square (*r*^*2*^) for each fitting curve is shown. Error bars around each point show standard errors of linear regression slope calculated from time course incubations with five bottles. Sampling depths for station PS2: Oxycline (90 m), ODZ top (120 m), and ODZ core (250 m); for station PS3: Oxycline (33 m), ODZ top (80 m), and ODZ core (160 m).

NO_2_^−^ oxidation was detected at several depths under various oxygen concentrations from an open ocean station (PS2) and a coastal station (PS3) (Fig. S1a). In most samples from the oxycline and top of the ODZ, NO_2_^−^ oxidation rates increased with increasing NO_2_^−^ concentration, consistent with Michaelis–Menten kinetics (Fig. 1). Half-saturation constants (*K*_*m*_) for these samples were similar to or slightly higher than those previously determined in low-nitrite OMZ [3] and non-OMZ [14] seawater, but much lower than those determined in cultures or other environments [14], confirming that the in situ assemblage of marine NOB is adapted to low-nitrite conditions. NO_2_^−^ oxidation rate from the ODZ core did not respond to NO_2_^−^ addition, suggesting saturation of the rate at ambient NO_2_^−^ concentrations (~2 µM, Fig. S1b and Table S1). O_2_ addition significantly increased the *K*_*m*_ of NO_2_^−^ oxidation at the ODZ top at station PS2 (Fig. 1f), indicating the co-existence of NOB with significantly different affinities for nitrite, as well as different oxygen preferences in this interface environment.

**Fig. 2:**
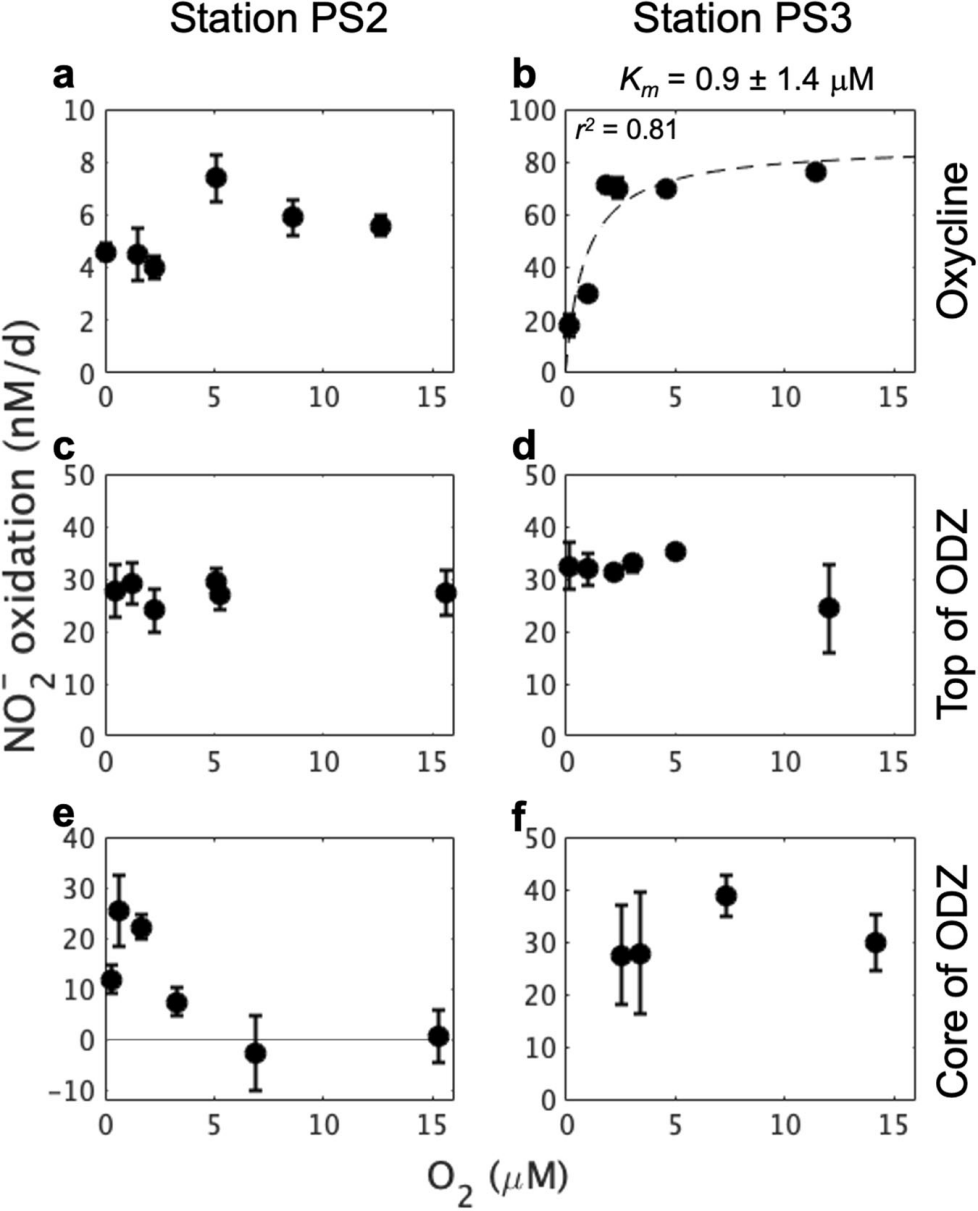
Response of NO_2_^−^ oxidation rates to O_2_ additions at stations PS2 and PS3. Samples were from oxycline (**a**, **b**), ODZ top (**c**, **d**), and ODZ core (**e**, **f**), respectively. Original O_2_ in all samples was purged with helium, and O_2_ concentrations shown on the *X*-axis were measured O_2_ concentrations in sample bottles after O_2_ additions. Dashed line means that *K*_*m*_ (half-saturation constant; see “Methods”) was not significantly different from zero. The adjusted R square (*r*^*2*^) for the fitting curve is shown. Error bars around each point show standard errors of the linear regression slope calculated from time course incubations with five bottles. Sampling depths for station PS2: Oxycline (93 m), ODZ top (120 m), and ODZ core (250 m); for station PS3: Oxycline (50 m), ODZ top (60 m), and ODZ core (160 m).

Unlike NO_2_^−^, O_2_ did not elicit simple Michaelis–Menten-like responses in NO_2_^−^ oxidation rate. O_2_ addition stimulated NO_2_^−^ oxidation rates in samples collected at oxycline depths (Fig. 2a, b), but had no effect on NO_2_^−^ oxidation rates at the top of the ODZ at either station (Fig. 2c, d). Notably, NO_2_^−^ oxidation rate at the ODZ core at station PS2 (Fig. 2e) appeared to be stimulated by 0.6 µM O_2_ but decreased to zero at higher O_2_ concentrations. To our knowledge, this is the first observation of full inhibition of NO_2_^−^ oxidation by O_2_ addition, and it occurred only in samples collected from the anoxic ODZ core. There was no significant response to added O_2_ at the ODZ core at station PS3 (Fig. 2f). Different responses to O_2_ of NO_2_^−^ oxidation at different depths imply different O_2_ tolerances as the basis of niche differentiation among OMZ NOB [8]. In particular, inhibition of NO_2_^−^ oxidation by O_2_ addition (>0.6 µM) at the ODZ core at station PS2 implies that the dominant NOB in these particular samples are adapted to anoxic environments. The lack of response to O_2_ at the top (Fig. 2c, d) or core of the ODZ (Fig. 2f) implies that diverse NOB with different O_2_ preferences co-existed and at least part of the in situ assemblage is something other than conventional obligate aerobic NOB.

### NO_2_^−^ oxidation and nitrite-oxidizing communities in the absence of O_2_ consumption

**Fig. 3:**
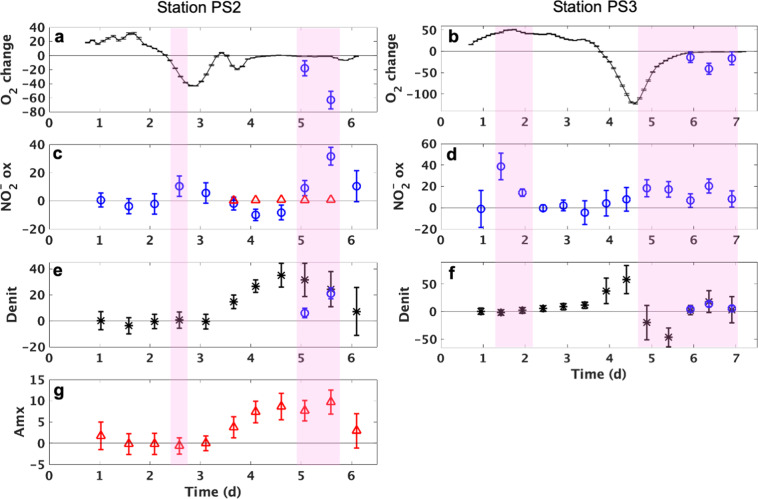
Nitrite oxidation and nitrogen loss processes determined in the core of the ODZ at stations PS2 and PS3. All rates are in nM/d or nM-N/d. **a**, **b** O_2_ increasing (positive values) and consumption (negative values) rates (black line) (nM/d) and estimated O_2_ consumption rates from nitrite oxidation if the nitrite oxidation had been aerobic (blue circles) (nM/d); (**c**, **d**) nitrite oxidation rates (blue) (NO_2_^−^ ox, nM-N/d) and estimated anaerobic nitrite oxidation rates from anammox (red); (**e**, **f**) denitrification rates (Denit, nM-N/d) (black stars) and estimated N_2_ production rates from nitrite disproportionation (blue circles); (**g**) anammox rates (Amx, nM-N/d). Error bars around each point show standard errors of linear regression slope calculated from time course incubations. Pink shade indicates the occurrence of nitrite oxidation (rates with error bars overlapping with zero line of *Y*-axis were not considered significant). O_2_ concentrations and excess nitrate or N_2_ used to calculate these rates are shown in Fig. S2.

The lowest O_2_ concentrations in the above incubations (0.3 µM at station PS2 and 2.6 µM at station PS3) with samples from the anoxic ODZ core (Fig. 2e, f) might still be high enough to support aerobic NO_2_^−^ oxidation. Therefore, week-long incubation experiments with samples from the anoxic ODZ cores were performed to determine whether NO_2_^−^ oxidation could occur independently of the O_2_ concentration. NO_2_^−^ oxidation occurred in samples collected from the anoxic ODZ core at both stations, where the measured O_2_ consumption rate was too slow to support the observed oxidation rates (Fig. 3a–d). O_2_ concentration increased during the first few days (Figs. 3a, b, S2a, b), probably due to release of trace O_2_ remaining in the rubber septa. Aerobic NO_2_^−^ oxidation occurred at day 2.5 at station PS2 and at 1.5 and 2 days at station PS3 (Fig. 3c, d). Then net O_2_ consumption commenced, which we attribute to microbial respiration and the complete deoxygenation of the septa. NO_2_^−^ oxidation occurred again (Fig. 3c, d) when ambient O_2_ was very low (i.e., 3 nM at station PS2 and 12 nM at station PS3, Fig. S2a, b) and O_2_ concentration stabilized after 4 days at station PS2 and 6 days at station PS3 (Fig. 3a, b). These later oxidation rates were much higher than could be accounted for by the contemporaneous O_2_ consumption rate (Fig. 3a, b) based on the stoichiometry of aerobic nitrite oxidation (Table S2). NO_2_^−^ oxidation occurred with and without O_2_, indicating either the co-existence of aerobic and anaerobic NOB at both stations, or a switch between metabolisms by the same microbes. Abiotic reactions can be ruled out (killed controls, see “Methods”). The maximum NO_2_^−^ oxidation rate (31.7 ± 6.3 nM d^−1^) at station PS2 was detected in the absence of O_2_ consumption and the maximum rate (38.64 ± 12.4 nM d^−1^) at station PS3 was aerobic. Together with evidence of O_2_ inhibition at station PS2 but not station PS3 (Fig. 2e, f), these results suggest that a larger proportion of NOB in the anoxic ODZ core at station PS2 were adapted to anaerobic conditions.

**Fig. 4:**
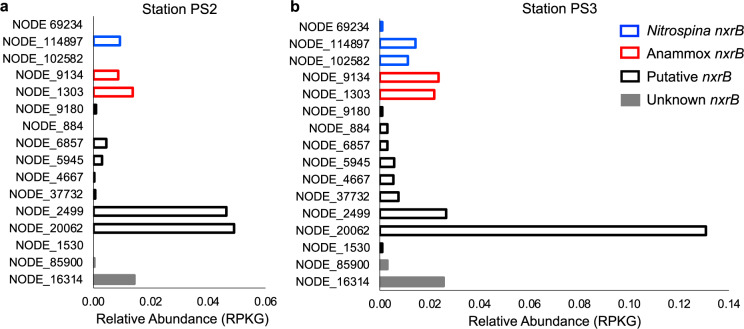
Relative abundances (RPKG) of previously identified OMZ *nxrB* genes in the core of the ODZ at ETNP stations PS2 and PS3. *Y*-axis shows IDs of *nxrB* genes. The presence of *nxrB* genes from clusters defined previously [8] shown in different colors indicates the presence of diverse nitrite oxidizers. Putative *nxrB* clustered between *Nitrospina nxrB* and Anammox *nxrB*, and Unknown *nxrB* did not fall into any known *nxrB* clusters.

To explore NOB communities in the anoxic ODZ cores at the two ETNP stations, we obtained metagenomes from the two ODZ core depths where the week-long incubation samples were collected. Two OMZ NOB “species” and several other putative NOB, recently identified in samples from the OMZ of the Eastern Tropical South Pacific (ETSP) [8] recruited ETNP metagenomic reads from this study, indicating that very similar NOB likely reside in the ETNP ODZ core samples where NO_2_^−^ oxidation was detected. NOB MAGs constructed here (ETNP PS2 MAG-11 and ETNP PS3 MAG-54) belong to the same species as MAG-2 from the ETSP based on their high average nucleotide identity (ANI) (99.5 and 99.1%, Table S3). We used the most complete draft genomes of the two novel OMZ NOB “species”, MAG-1 and MAG-2 from the ETSP (Table S4), to estimate their relative abundance in the total microbial communities in the ODZ cores at our two ETNP stations by mapping new sequencing reads from the ETNP to the two ETSP MAGs. Relative abundance of NOB, expressed as RPKG (reads per kilobase per genome equivalent) [15], varied between the two stations (Fig. 4), but MAG-2 was always more abundant than MAG-1 (Table S5). Based on the relative enrichment of MAG-1 at oxic–anoxic interfaces and MAG-2 at anoxic ODZ cores, MAG-1 was proposed as a microaerophile while MAG-2 might be an anaerobic specialist [8]. A terminal oxidase with high affinity for O_2_ was encoded by MAG-1 but was missing in MAG-2 [8]. Although anaerobic metabolisms were not confirmed in MAG-2, MAG-1 and MAG-2 may have different O_2_ sensitivities. The relative abundance of MAG-2 was 9.8-fold higher than MAG-1 at station PS2, but only 1.9-fold higher at station PS3 (Table S5), consistent with the greater relative dominance of apparently anaerobic NO_2_^−^ oxidation (Fig. 3c) and the inhibition of NO_2_^−^ oxidation by O_2_ (Fig. 2e) at station PS2. We also estimated the relative abundance (RPKG) of anammox and other (putative) NOB by mapping ETNP metagenomic reads obtained in this study to previously identified OMZ *nxrB* (nitrite oxidoreductase) gene sequences (Fig. 4). The two most abundant *nxrB* genes at both stations belonged to the putative *nxrB* cluster and they cannot be linked to any known NOB or anammox. The abundance of these unknown genes, representing potentially unknown metabolisms, suggests that other undiscovered microbes could be even more important than the known species and MAGs in the metabolism of nitrite in the ODZ.

### Potential mechanisms corresponding to NOB niche differentiation along redox gradients

NO_2_^−^ oxidation in distinct redox layers (i.e., the top of the ODZ and the core of the ODZ) can be explained by different mechanisms (Fig. S3). NOB respire O_2_ in order to oxidize nitrite in the oxic layer. At the top of the ODZ, the interface where O_2_ is usually undetectable, transient intrusions [16] could support NO_2_^−^ oxidation. When O_2_ is undetectable, cryptic oxygen cycling (i.e., supported by in situ O_2_ production in the deep chlorophyll maximum, attributed to abundant *Prochlorococcus*) [12] could support the in situ rate, even though all incubations in this study were in the dark. Some of the NOB at the top of the ODZ were found to have very high affinity for O_2_, higher than their nitrification partner ammonia oxidizers in the same environment [5]. Thus, these NOB could use the trace amount of O_2_ which is available at this oxic–anoxic interface. Alternatively, iodate is proposed as a possible oxidant for NO_2_^−^ [17] and iodate flux from the oxic layer is possible at the ODZ top.

In the ODZ core, several mechanisms must be considered: (a) Neither O_2_ nor iodate is available (Fig. S3) in the ODZ core. Iodate and iodide concentration measurements made in the same month or year that these rates were measured show that iodate was usually depleted in the ETNP ODZ core [18]. Iodate concentrations are nearly constant near the secondary nitrite maximum inside ODZs [18]; thus iodate flux is not sufficient to support observed NO_2_^−^ oxidation rates. In addition, iodate reduction was detected in oxyclines but was usually below detection limit in anoxic ODZs when iodate was added in the presence of nitrite [19], which indicates that microbes in those samples could not use iodate to oxidize nitrite.

(b) Transient intrusion of O_2_ has been reported [20] but is very rare in the core [16]. Anaerobic NO_2_^−^ oxidation observed in incubation experiments above suggests that this reaction could occur without O_2_.

(c) Chlorophyll concentration was very low (Fig. S1b) in ODZ samples and the ODZ core was dark, so photosynthesis could not support an O_2_ flux for nitrite oxidation in situ or in the vials.

(d) The oxidation of NO_2_^−^ by Mn^4+^ or Fe^3+^ is thermodynamically favored only at very low pH (<6) [21]. Even if enzymatic oxidation by these metals is possible at higher pH, total dissolved Mn (0–5 nM) and Fe (0–1 nM) concentrations are too low in OMZs [22, 23] to support the observed nitrite oxidation rates.

(e) Anammox bacteria anaerobically oxidize NO_2_^−^ to NO_3_^−^ while simultaneously using NH_4_^+^ to reduce NO_2_^−^ to N_2_ [24]. Based on known reaction stoichiometry (Table S2) [24], the anammox rates measured in the same bottles where we detected NO_2_^−^ oxidation rates at station PS2 (Fig. 3g) accounted for 7% and 2% of the measured anaerobic NO_2_^−^ oxidation rates at days 5 and 5.5, respectively (Fig. 3c). At station PS3, anammox was not detected. Thus, anammox alone could not explain anaerobic NO_2_^−^ oxidation.

(f) The reversible nitrite oxidoreductase enzyme [25] has been proposed to catalyze the bidirectional exchange between NO_2_^−^ and NO_3_^−^, independent of net concentration change [26, 27]. This “futile” cycle could lead to NO_2_^−^ and NO_3_^−^ isotopic fractionation without generating energy to support cell growth. Thus, this process alone might explain the isotope enrichment in NO_3_^−^ from labeled ^15^NO_2_^−^ (Fig. S2c, d), but cannot explain inhibition of NO_2_^−^ oxidation by O_2_ at station PS2 (Fig. 2e), the presence of NOB at both stations (Fig. 4), transcriptionally active NOB in the ODZs of ETNP and ETSP OMZs (Fig. S4), or the increasing number of nitrite oxidoreductase enzymes with decreasing O_2_ in the OMZ [28]. This suggests that in situ NO_2_^−^ oxidation at both stations occurs by some energy generating mechanism, at least some of the time, to support NOB growth (Fig. S3).

(g) NO_2_^–^ disproportionation

1 $$5{\rm{NO}}_{2}^{-} \,+\, 2{\rm{H}}^{+} \rightarrow {\rm{N}}_{2} \,+\, 3{\rm{NO}}_{3}^{-} \,+\, {\rm{H}}_{2}{\rm{O}},$$

was suggested as a potential explanation for nitrite oxidation carried out by NOB adapted to anoxic environments [6, 29]. This reaction is thermodynamically favorable [30, 31] although it has not been detected in nature. Disproportionation, if confirmed, constitutes a third pathway of nitrogen loss in addition to anammox and denitrification. Because the two N atoms in N_2_ must come from NO_2_^−^ during both NO_2_^−^ disproportionation and denitrification, the isotope signatures of N_2_ produced from both pathways in the tracer incubations would be identical. Thus, previously measured “denitrification” using stable isotope tracer experiments might actually be a combination of canonical denitrification plus NO_2_^−^ disproportionation. Based on stoichiometry (Eq. (1)), N_2_ produced from NO_2_^−^ disproportionation could be responsible for 20–100% of the measured rate of N_2_ production from NO_2_^−^ based on measured N_2_ production and NO_2_^−^ oxidation rates here (Fig. 3e, f).

OMZ NOB may have the potential to carry out NO_2_^−^ disproportionation by coupling NO_2_^−^ oxidation and NO_2_^−^ reduction (Table S6). The only gene required for this process that is missing from the ODZ MAGs is a NO dismutase gene (*nod*). No sequences related to the *nod* of *Candidatus* Methylomirabilis oxyfera [32] were found in the two ETSP NOB MAGs [8] or the two ETNP MAGs. Unidentified *nod* distinct from that of *Ca*. Methylomirabilis oxyfera might be present but cannot be identified due to the lack of *nod* references. Alternatively, chlorite dismutation following chlorate reduction to chlorite by NXR [33] could supply internal O_2_, although chlorate and chlorite concentrations in the ocean are vanishingly small. Chlorite dismutase (Cld) was found to be functional in *Nitrobacter* NOB and *Nitrospira* NOB [34, 35], and was proposed to provide O_2_ for NOB in hypoxic conditions [34]. DNA sequences encoding Cld-like proteins were present in NOB MAGs from the ETSP OMZ [8] and ETNP PS2 MAG-11 and ETNP PS3 MAG-54 despite the low MAG quality of ETNP MAGs. However, arginine173, which was proposed as a marker for Cld activity [36], was replaced by leucine in MAG-1 and MAG-2 (Fig. S5a). Although the same position of Cld-like proteins was not included in the short Cld-like protein sequences (only 120 aa) encoded by ETNP MAGs, their high similarity to the Cld-like protein of MAG-2 (Fig. S5b) suggests that the chance of finding arginine173 in Cld-like proteins of ETNP NOB MAGs was low. Thus, the primary function of Cld-like proteins in all *Nitrospina*-like OMZ NOB needs to be further confirmed by experiments. NOB from other genera have surprisingly versatile metabolic capabilities [6, 9, 25]. OMZ NOB may also use other yet to be discovered metabolisms to survive in anoxic waters.

### Implications for the marine nitrogen budget from a biogeochemical model

**Fig. 5:**
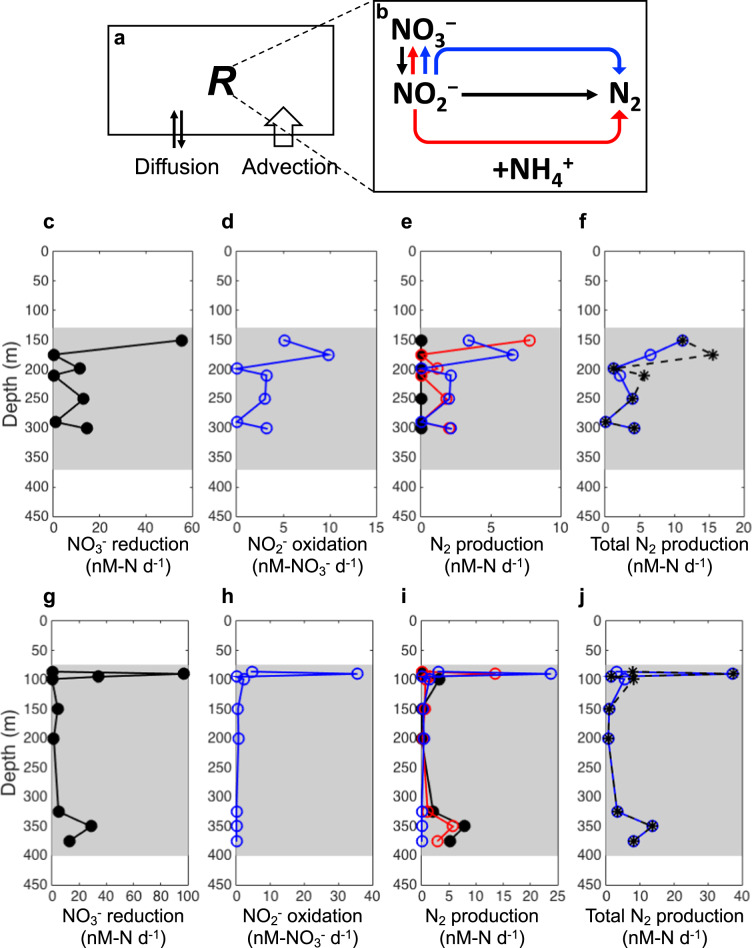
A schematic of an inverse 1-D model and modeled nitrogen cycling rates. **a**, **b** A schematic of physical processes and net biochemical production or consumption (*R*) that affect concentrations of inorganic nitrogen compounds (**a**), and nitrogen cycle processes resulting in net production or consumption (*R*) in the anoxic core of the ODZ represented in the model (**b**). Black arrows: nitrate reduction and denitrification (nitrite reduction to N_2_). Red arrows: anammox. Blue arrows: nitrite oxidation through disproportionation. **c–j** Model results: (**c**, **g**) Modeled rates of nitrate reduction; (**d**, **h**) Nitrate production from nitrite disproportionation; (**e**, **i**) Denitrification (black), anammox (red), N_2_ production from nitrite disproportionation (blue); (**f**, **j**) Total N_2_ production when including nitrite oxidation through disproportionation (blue) and excluding nitrite oxidation in the model (black) at stations BB1 (**c**–**f**) and BB2 (**g–j**) in the ETSP OMZ. Shaded areas indicate the location of the anoxic ODZ.

We added NO_2_^−^ disproportionation to an established inverse model [37] to quantitatively test the importance to the nitrogen budget of this newly proposed pathway (Fig. 5a, b). This model simulated nitrogen cycling rates in ODZs of the ETSP OMZ, where measured concentrations [38] and natural abundance isotope data [37] of nitrogen compounds were available (Table S7). All rates simulated in the revised model (Fig. 5c–j), especially NO_2_^−^ reduction and NO_2_^−^ oxidation through NO_2_^−^ disproportionation (up to 36 nM-NO_3_^−^ d^−1^), were a better match (i.e., the same magnitude) for the measured rates (at the same time and location of the collection of model input data, shown in Fig. 8 in Peters et al. 2016 [37]) than the rates simulated in the original model [37], which were an order of magnitude larger than the measured rates. The simulated total nitrogen loss was similar or much lower (by up to 62%) when NO_2_^−^ disproportionation is included in anoxic ODZs (Fig. 5f, j) because the light NO_2_^−^ and heavy NO_3_^−^ in the ODZ can be attributed to the inverse isotopic fractionation of NO_2_^−^ oxidation [39, 40] in addition to the fractionation of conventional nitrogen loss processes. Although the mechanisms for NO_2_^−^ oxidation in anoxic waters remain to be experimentally verified, the modeling results here indicate the necessity to consider microbial niche differentiation when simulating reactions in distinct redox conditions.

NO_2_^−^ oxidation is a critical pivot in the nitrogen cycle, constraining the proportion of nitrogen retention and affecting total estimates of nitrogen loss. Most current models use a fixed positive O_2_ threshold to determine when and where NO_2_^−^ oxidation occurs in OMZs, and one parameterization to characterize its kinetics. Our results, however, demonstrate that NO_2_^−^ oxidation occurred throughout the redox gradient of OMZs, and that substrate kinetics, O_2_ responses and potential mechanisms of NO_2_^−^ oxidation differed among depths, potentially due to diverse NOB occupying distinct niches. Marine suboxic zones are predicted to expand over the next 130 years due to global climate change [41]. Dynamic regions such as coastal upwelling zones are subject to seasonal nutrient and oxygen fluctuations. Thus, responses of NO_2_^−^ oxidation to NO_2_^−^ and O_2_ determined here should be applied to other systems and could improve predictions of both long- and short-term nitrogen budgets in a changing ocean. The finding of nitrite oxidation occurring without O_2_ but inhibited by O_2_ addition in ODZs calls for follow-up research, especially the isolation of novel NOB from anoxic environments.

## Methods

### Experimental site

Seawater samples were collected from two stations (Fig. S1a, offshore OMZ station PS2 and coastal OMZ station PS3) in the ETNP in March and April 2018 on board R/V Sally Ride (Cruise ID: SR 1805). NO_2_^−^ kinetics and O_2_ effects experiments (≤1 day incubation) were performed at the oxycline, the oxic/anoxic interface (top) of the ODZ and the core of the ODZ at each station (Fig. S1b and Table S1). Long incubations (6–7 days) were performed at the core of the ODZ at both stations to investigate nitrogen reaction rates at very low O_2_ concentrations.

### Sampling and incubation experiments for NO_2_^−^ kinetics and O_2_ effects

Twelve 30-L Niskin bottles on a rosette with a conductivity–temperature–depth (CTD) profiler were used to collect seawater while recording in situ O_2_ concentration, temperature, pressure, salinity, and chlorophyll fluorescence. O_2_ concentration at selected ODZ depths was measured by STOX sensor (on the CTD profiler) with detection limit of 10 nM [42]. NO_2_^−^ concentrations were measured by standard spectrophotometric methods onboard. O_2_ and NO_2_^−^ concentrations were used to select sampling depths. NO_3_^−^ and NO_2_^−^ concentrations in incubation samples were measured on a chemiluminescence NO/NOx Analyzer (Teledyne API, San Diego, CA, USA) in the lab.

Seawater was collected from Niskin bottles into 60 mL air-tight serum bottles after overflowing three times in order to minimize O_2_ contamination. Serum bottles were sealed with rubber septa and aluminum seals ensuring the absence of bubbles inside bottles. Septa were deoxygenated in anaerobic chambers with three cycles of vacuum/helium flushing over a period of 1 month prior to the cruise. A helium headspace was created for each sample collected from anoxic depths (the top and the core of the ODZ), and then samples were flushed with helium for at least 15 min to remove O_2_ that might have been introduced during sampling.

To determine a single NO_2_^−^ oxidation rate, a set of five serum bottles amended with ^15^NO_2_^−^ tracer was incubated on board at 12 °C in the dark. Incubations were terminated in time series (one bottle at day zero (T_0_), two at 0.5 day and two at 1 day) by adding 0.2 mL of saturated ZnCl_2_. The T_0_ bottles served as killed controls for both tracer contamination and abiotic reactions. The observed temporal changes in isotopic signals that occurred in the live samples over a few days in the time courses would not be detected if abiotic reactions were taking place during the >3 months that elapsed before all samples were measured on the mass spec.

In the lab, NO_2_^−^ was removed from samples using sulfamic acid, and NO_3_^−^ in serum bottles was converted into N_2_O using the denitrifier method [43, 44]. Both concentration and isotopic composition of N_2_O were measured on a mass spectrometer (Delta V^plus^, Thermo Fisher Scientific, Waltham, MA, USA) for calculating nitrite oxidation rate from the linear regression of the five nitrate concentrations at the three time points as previously described [3]. NO_2_^−^ kinetics of NO_2_^−^ oxidation were determined by measuring NO_2_^−^ oxidation rates under different ^15^NO_2_^−^ tracer concentrations (0.5–13.8 μM). For responses of NO_2_^−^ oxidation to O_2_, different amounts (0, 0.2, 0.5, 1, 2, and 5 mL) of O_2_ saturated seawater collected from the same Niskin bottle were added into serum bottles to achieve different O_2_ concentrations. O_2_ concentrations in serum bottles were monitored by optical oxygen sensors with a detection limit of 62.5 nM (PyroScience GmbH, Aachen, Germany).

### Half-saturation constant

Half-saturation constant (*K*_m_) is the nitrite concentration at which nitrite oxidation rate (*V*) equals half of the potential maximum rate (*V*_m_). The curve fitting tool in MATLAB_R2015a was used to fit the Michaelis–Menten model (Eq. (2)) to determine *K*_m_.

2 $${{V}} \,=\, {{V}}_{\mathrm{m}} \,\times\,\left[ {{\mathrm{NO}}_2^-} \right]{\mathrm{/}}\left( {\left[ {{\mathrm{NO}}_2^-} \right] \,+\, {{K}}_{\mathrm{m}}} \right).$$

### Long incubation experiments

Longer (≥6 days) incubations were performed in 12-mL exetainers. Seawater from the core of the ODZ (250 m at station PS2, 160 m at station PS3) was sampled into 320 mL ground glass-stoppered bottles, which were immediately transferred into a N_2_ flushed glove bag. ^15^NO_2_^−^ was added into these bottles to reach final concentrations of 7.24 and 8.01 µM at stations PS2 and PS3, respectively. ^15^NO_2_^−^ labeled seawater was aliquoted into exetainers and capped within the glove bag. The septa had been stored under helium for at least 1 month. Exetainers were purged with helium for 5 min. Every 12 h, microbial activity in triplicate exetainers was terminated by adding 0.05 mL of 50% w/v ZnCl_2_. In the lab, N_2_ produced in exetainers was measured on a mass spectrometer (Europa Scientific 20–20, Crewe, UK) [45]. Denitrification and anammox rates were computed from linear regression of N_2_ produced at three time points. ^15^NO_3_^−^ was measured (as described above) in the same exetainers, and NO_2_^–^ oxidation rates were computed from linear regression of NO_3_^−^ produced at three time points. O_2_ was monitored throughout the incubations in parallel exetainer vials using LUMOS sensors [12, 13]. Each O_2_ production or consumption rate was determined by linear regression of O_2_ concentrations at 32 time points. One sensing spot, glued inside an exetainer vial, allowed the optode to measure O_2_ concentrations every 5 min from the outside. Detection limit and resolution of LUMOS sensors was ≈1 nM.

### DNA sampling, extraction, sequencing, metagenomics, and metatranscriptomics analysis

Particulate DNA samples were collected by filtration onto 0.22 µm Sterivex filters from the ODZ core samples (250 m at station PS2, 160 m at station PS3). DNA was extracted using the modified plant tissue protocol (All Prep DNA/RNA Mini Kit, Qiagen, Valencia, CA, USA), and subjected to paired-end sequencing on an Illumina MiSeq to generate over 10 million read pairs for each sample by the Genomics Core Facility of Lewis-Sigler Institute for Integrative Genomics at Princeton University. Quality control of raw reads was performed by BBDuk (DOE Joint Genome Institute, Walnut Creek, CA, USA), and assembled into contigs using metaSPAdes v3.12.0 [46] with specified options (-k 21, 33, 55, 77, 99, 127 -m 500). Metagenome-assembled genomes (MAGs) were constructed using MetaBAT v2.12.1 [47] with default (sensitive) mode and contigs longer than 1500 bp. The quality of MAGs was determined by checkM [48]. Taxonomy of MAGs was predicted by GTDB-tk [49]. ETNP PS2 MAG-11 from the ODZ core at station PS2 and ETNP PS3 MAG-54 from the ODZ core at station PS3 were identified as *Nitrospina*-like NOB. The taxonomy of these two NOB MAGs (ETNP PS2 MAG-11 and ETNP PS3 MAG-54) was further confirmed by comparing them with known OMZ NOB MAGs (MAG-1 and MAG-2) [8] using ANI. ANI between MAGs was assessed using enveomics [50]. MAG-2 [8], ETNP PS2 MAG-11 and ETNP PS3 MAG-54 belong to the same species (threshold for species: ANI ≥95%) based on their ANI values (Table S3). Considering the low completeness of MAG-11 and the high contamination of MAG-54 constructed from the two new metagenomes here (Table S4), we decided to use MAG-2 as the representative for this NOB species.

We estimated the relative abundance and the transcriptional activity of the two known OMZ NOB species in different oceanic regions (including the two ETNP stations in this study) by mapping metagenomes and metatranscriptomes from this and other studies to MAG-1 and MAG-2. The relative abundance of MAG-1 or MAG-2 at stations PS2 and PS3 in the ETNP was calculated as RPKG (the number of metagenomic reads obtained in this study mapped to a MAG per MAG length (kb) per genome equivalents) [15]. Genome equivalents were estimated using MicrobeCensus v1.1.1 [15]. The transcriptional activity of MAG-1 or MAG-2 in ETNP and ETSP OMZs was assessed by mapping published metatranscriptomic reads from the ETNP (PRJNA263621) [51] and the ETSP (SRA023632.1) [52] to MAG-1 and MAG-2. The relative abundance of RNA in Fig. S4 was calculated as the number of metatranscriptomic reads mapped to a MAG divided by the number of total reads. Mapping was performed by Bowtie2 [53] using “very-sensitive” mode, and only reads with a mapping quality above 20 were included as mapped reads.

In order to explore the possibility of the presence of other NOB in the ODZ core at stations PS2 and PS3, we also estimated the relative abundance of other (putative) NOB using their marker gene, *nxrB* (nitrite oxidoreductase). First, we downloaded previously identified (putative) *nxrB* sequences from the ETSP OMZ [8]. Then, we color coded the genes in Fig. 4 based on previously defined clusters in a phylogenetic tree (see Fig. 3 in [8]): the *Nitrospina* cluster (blue) contains *nxrB* grouped with cultured marine NOB, *Nitrospina gracilis*. Based on BLASTp search results, amino acid sequence identities between all the OMZ *nxrB* in this cluster and that of *Nitrospina gracilis* were 96.71%, 96.71%, and 96.87% for NODE_69234, NODE_114897, and NODE_102582, respectively. Only this cluster is associated with known NOB, and the nitrite oxidation capacity of all the other genes associated with *nxrB* needs to be confirmed. The anammox cluster (red) contains anammox *nxrB* sequences. The putative cluster (black) contains *nxrB* grouped with microbes in which nitrite oxidation capacity has not been proven. This putative cluster fell between known NOB and anammox, and was implied to represent unidentified NOB [54]. The last cluster is called unknown *nxrB* (gray) because neither their phylogeny nor function can be determined based on their distant relationship with known NOB or anammox *nxrB*. Finally, we mapped the ETNP metagenomic reads obtained in this study to each *nxrB* gene. Relative abundance of *nxrB* genes was also expressed as RPKG: relative abundance of *nxrB* gene = (number of mapped reads to a certain *nxrB* gene)/(length of this *nxrB* in kb)/(genome equivalents).

To explore the potential metabolisms of NOB in anoxic ODZ cores, we searched for chlorite dismutase and NO dismutase (*nod*) genes in the NOB MAGs. First, protein-coding sequences in the two new NOB MAGs obtained here (ETNP PS2 MAG-11 and ETNP PS3 MAG-54) were predicted by Prodigal v2.6.3 [55]. The protein-coding sequences were annotated by the best BLASTp hits against the nr protein database. DNA sequences encoding Cld were identified in both ETNP NOB MAGs. Predicted Cld amino acid sequences encoded by ETNP MAGs were too short (120 aa) to be compared to Cld of *Candidatus* Nitrospira defluvii, but they only had one mismatch with Cld amino acid sequences of MAG-2 from the ETSP OMZ based on MUSCLE alignment using MEGA 7 software (Fig. S5b). Thus, we looked for the arginine173, the Cld activity marker, in longer Cld sequences of MAG-1 and MAG-2 by aligning their Cld with the Cld of *Candidatus* Nitrospira defluvii using MUSCLE in MEGA 7 software. Since *nod* was not found in the ETNP MAGs via annotation and was not reported in MAG-1 and MAG-2 in the previous study [8], gene search (i.e., searching *nod* against NOB MAGs) was performed using Hidden Markov Models by HMMER3 [56]. Reference sequences of the search included a *nod* sequence retrieved from *Candidatus* Methylomirabilis oxyfera [32] genome (accession numbers FP565575.1), and three environmental *nod* sequences (accession numbers: KX364450.1, KX364454.1, and KU933965.1). Search queries were the two ETNP MAGs and two ETSP MAGs in Table S3.

### Estimation of NO_2_^−^ oxidation through disproportionation using an inverse isotope model

We simulated the distribution of NO_2_^−^ oxidation rates via disproportionation using a 1-D inverse isotope model [37] in the anoxic ODZ core from two stations in the ETSP OMZ (Fig. S6), where the complete suite of isotope and rate data have been previously published (Table S7). Briefly, the net biochemical production or consumption rate of each nitrogen compound (*R*_*14Ammonium*_, *R*_*14Nitrite*_, *R*_*14Nitrate*_, *R*_*15Nitrite*_, and *R*_*15Nitrate*_) was balanced by vertical diffusion and advection at steady state, and then five equations were used to balance measured ^14^NH_4_^+^, ^14^NO_2_^−^, ^15^NO_2_^−^, ^14^NO_3_^−^, and ^15^NO_3_^−^ concentrations (Eqs. (3–7)). The exclusion of horizontal processes was justified [37] since the model was not run all the way to the surface, and using a constant vertical advection term does not violate continuity of the model. *F* is the rate of each nitrogen cycle process represented in this model (Fig. 5a, b). We modified the previous model by replacing canonical NO_2_^−^ oxidation with NO_2_^−^ disproportionation and using the recently determined stoichiometry of nitrate production by anammox from 0.3 to 0.16 [24]. Since OMZ NOB genomes encode nitrite oxidoreductase (catalyzes NO_2_^−^→NO_3_^−^) and nitrite reductase (catalyzes NO_2_^−^→NO), we assumed that the fractionation factor of nitrate production from nitrite by disproportionation is the same as by canonical nitrite oxidation (*α*_*Dis*_ = *α*_*Nxr*_) based on the close clustering between OMZ NOB *nxr* and aerobic NOB (*Nitrospina gracilis*) *nxr* [8], and the fractionation factor of N_2_ produced by disproportionation is the same as by denitrification (*α*_*DisN2*_ = *α*_*Nir*_). Rates of NO_2_^−^ disproportionation (*F*_*Dis*_), NO_3_^−^ reduction (*F*_*Nar*_), denitrification (*F*_*Nir*_), and anammox (*F*_*Amx*_) were solved from the equations by the nonnegative least squares optimization routine (lsqnonneg) in MATLAB_R2015a as described previously [37].

3 $$R_{14Ammonium} \,=\, 0.11\,\times {\,}^{14}F_{Nir} \,+\, 0.07\,\times {\,}^{14}F_{Nar} \,- {\,}^{14}F_{Amx},$$

4 $$R_{14Nitrite} = - \,^{14}F_{Nir} \,+\, {\,}^{14}F_{Nar} \,-\, (1 \,+\, c) \,\times\,{\,}^{14}F_{Amx} \\ -\, \left( {5/3} \right) \,\times\,{\,}^{14}F_{Dis},$$

5 $$R_{14Nitrate} \,=\, - ^{14}F_{Nar} \,+\, c \,\times {\,}^{14}F_{Amx} \,+\, {\,}^{14}F_{Dis},$$

6 $$R_{15Nitrite} = 	-\!\! {\,}^{14}F_{Nir}/\alpha_{Nir} \,\times\,\left( {\left[ {{\,}^{15}{\rm{NO}}_{2}^{-}} \right]/\left[ {{\,}^{14}{\rm{NO}}_{2}^{-}} \right]} \right) \\ 	 +\, {\,}^{14}F_{Nar}/\alpha_{Nar}\,\times\,\left( {\left[ {{\,}^{15}{\rm{NO}}_{3}^{-}} \right]/\left[ {{\,}^{14}{\rm{NO}}_{3}^{-}} \right]} \right)\\ 	 -\, {\,}^{14}F_{Amx}/\alpha_{Amx}\,\times\,\left( {\left[ {{\,}^{15}{\rm{NO}}_{2}^{-}} \right]/\left[ {{\,}^{14}{\rm{NO}}_{2}^{-}} \right]} \right) \\ 	-\, c \times \!{\,}^{14}F_{Amx}/\alpha_{NxrAmx}\,\times\,\left( {\left[ {{\,}^{15}{\rm{NO}}_{2}^{-}} \right]/\left[ {{\,}^{14}{\rm{NO}}_{2}^{-}} \right]} \right)\\ 	 -\, {\,}^{14}F_{Dis}/\alpha_{Dis}\times\left( {\left[ {{\,}^{15}{\rm{NO}}_{2}^{-}} \right]/\left[ {{\,}^{14}{\rm{NO}}_{2}^{-}} \right]} \right) \\ 	-\, \left( {2/3} \right) \!\times \! {\,}^{14}F_{Dis}/\alpha_{DisN2} \!\times \left(\! {\left[ {{\,}^{15}{\rm{NO}}_{2}^{-}} \right]/\left[ \!{{\,}^{14}{\rm{NO}}_{2}^{-}} \right]} \!\right),$$

7 $$R_{15Nitrate} = 	-\!\! {\,}^{14}F_{Nar}/\alpha_{Nar} \,\times\,\left( {\left[ {{\,}^{15}{\rm{NO}}_{3}^{-}} \right]/\left[ {{\,}^{14}{\rm{NO}}_{3}^{-}} \right]} \right) \\ 	+\, c \times \!{\,}^{14}F_{Amx}/\alpha_{NxrAmx}\,\times\,\left( {\left[ {{\,}^{15}{\rm{NO}}_{2}^{-}} \right]/\left[ {{\,}^{14}{\rm{NO}}_{2}^{-}} \right]} \right)\\ 	 +\, ^{14}F_{Dis}/\alpha_{Dis}\,\times\,\left( {\left[ {{\,}^{15}{\rm{NO}}_{2}^{-}} \right]/\left[ {{\,}^{14}{\rm{NO}}_{2}^{-}} \right]} \right).$$

## Supplementary information

Supplementary materials

## Data Availability

NOB MAG-1 and MAG-2 from ETSP metagenomes were submitted to NCBI under BioSample accession numbers SAMN10411459 (MAG-1) and SAMN10411419 (MAG-2). Metagenomic reads from the core of the ODZ at stations PS2 and PS3 were deposited to NCBI under the SRA accession number PRJNA505148.

## Code availability

Code for the 1-D model is available at https://github.com/xinsun12/AnaerobicNitriteOxidation.
