# Correction: Microbial niche differentiation explains nitrite oxidation in marine oxygen minimum zones

**DOI:** 10.1038/s41396-021-01032-7

**Published:** 2021-07-06

**Authors:** Xin Sun, Claudia Frey, Emilio Garcia-Robledo, Amal Jayakumar, Bess B. Ward

**Affiliations:** 1 Department of Geosciences, Princeton University, Princeton, NJ USA; 2 Department of Biology, Calle Republica Saharaui, University of Cadiz, Puerto Real, Cadiz Spain; 3 Present Address: Department of Environmental Science, University of Basel, Basel, Switzerland

**Keywords:** Biogeochemistry, Water microbiology, Microbial ecology

Correction to: *The ISME Journal* 10.1038/s41396-020-00852-3, published online 06 January 2021

After publication of this article, the authors realized that proteins encoded by the OMZ NOB MAGs were incorrectly annotated as chlorite dismutase (Cld). They are homologs of Cld, and they should be called Cld-like proteins. Key residues for Cld are missing in these proteins and their chlorite dismutation activity is not expected. Thus, we correct ‘Cld’ into ‘Cld-like proteins’ for OMZ NOB MAGs.

The authors acknowledge Dr. Tyler Barnum for pointing out this issue.

The original article has been corrected.

