# Supplementary material for: Microbial niche differentiation explains nitrite oxidation in marine oxygen minimum zones
Source: ISME J. 2021 Jan 6;15(5):1317–29. doi: 10.1038/s41396-020-00852-3 (PMC8114937; doi:10.1038/s41396-020-00852-3)
Supplement: Supplementary file 1 — Supplementary materials [file 41396_2020_852_MOESM1_ESM.pdf]

Supporting information for

Microbial Niche Differentiation Explains Nitrite Oxidation in Marine Oxygen Minimum Zones

Xin Sun, Claudia Frey, Emilio Garcia-Robledo, Amal Jayakumar, Bess B. Ward
Correspondence to: xins@princeton.edu

Figures

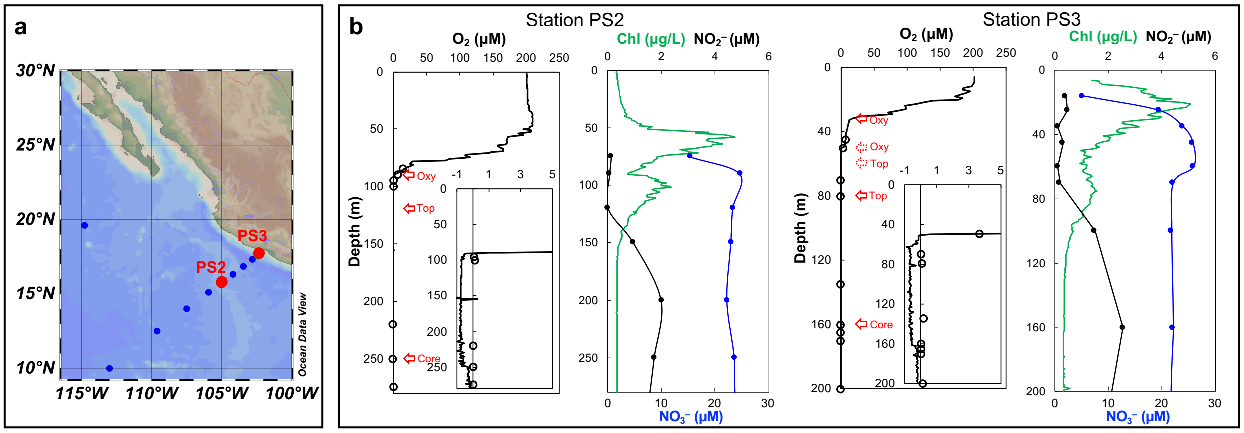

Figure S1. Locations and conditions of the two sampling stations. (**a**) Locations of the two stations (PS2 and PS3). (**b**) oxygen (O_2), chlorophyll (Chl), nitrite and nitrate profiles at these stations. Red arrows indicate the depths where samples were collected for kinetics and O_2 additions experiments. Station PS3 had a dynamic oxycline. In order to capture the oxic-anoxic interface, sampling depths for O_2 additions experiments differed from the depths for kinetics at Station PS3. The two dashed red arrows at station PS3 reflect the different sampling depths for O_2 additions experiments. The features targeted at each depth are shown as Oxy (oxycline), Top (top of the ODZ) and Core (core of the ODZ). Open black circles indicate O_2 measured in situ by STOX sensor.

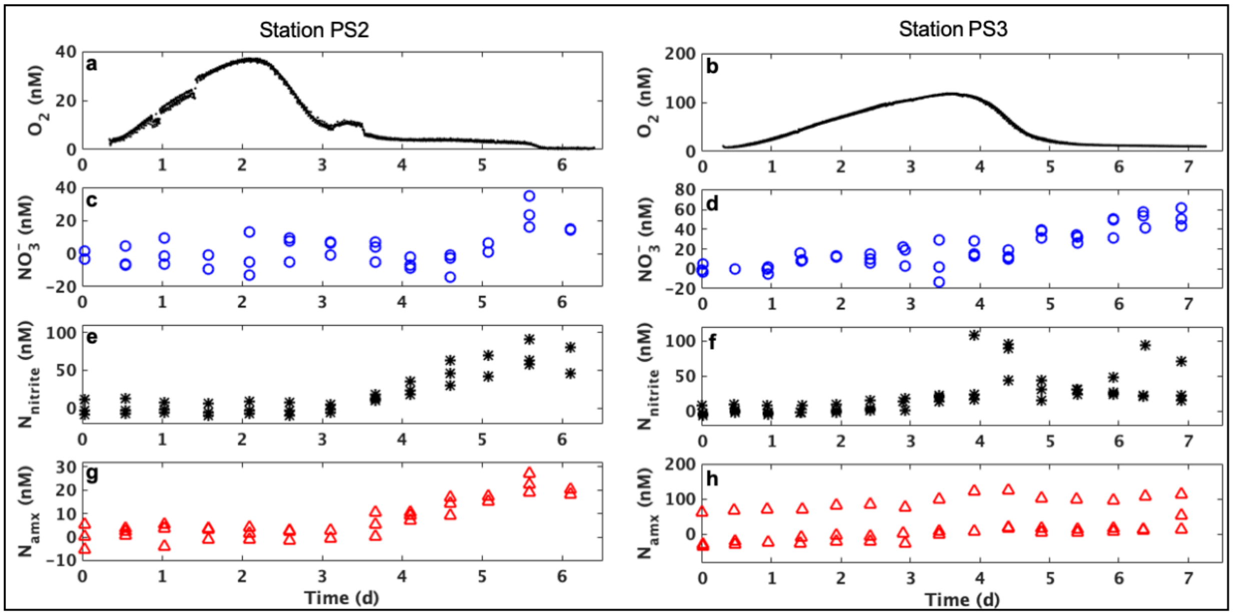

Figure S2. Changes in O_2 concentrations and excess ^{15}N-nitrate or N_2 used for rate calculation in Fig. 3. Changes in oxygen (**a, b**); excess nitrate produced from nitrite oxidation (**c, d**); excess nitrogen produced from nitrite (i.e. canonical denitrification + potentially nitrite disproportionation) (**e, f**); anammox (excess nitrogen from nitrite and ammonium) (**g, h**) over time in the core of the ODZ at stations PS2 and PS3.

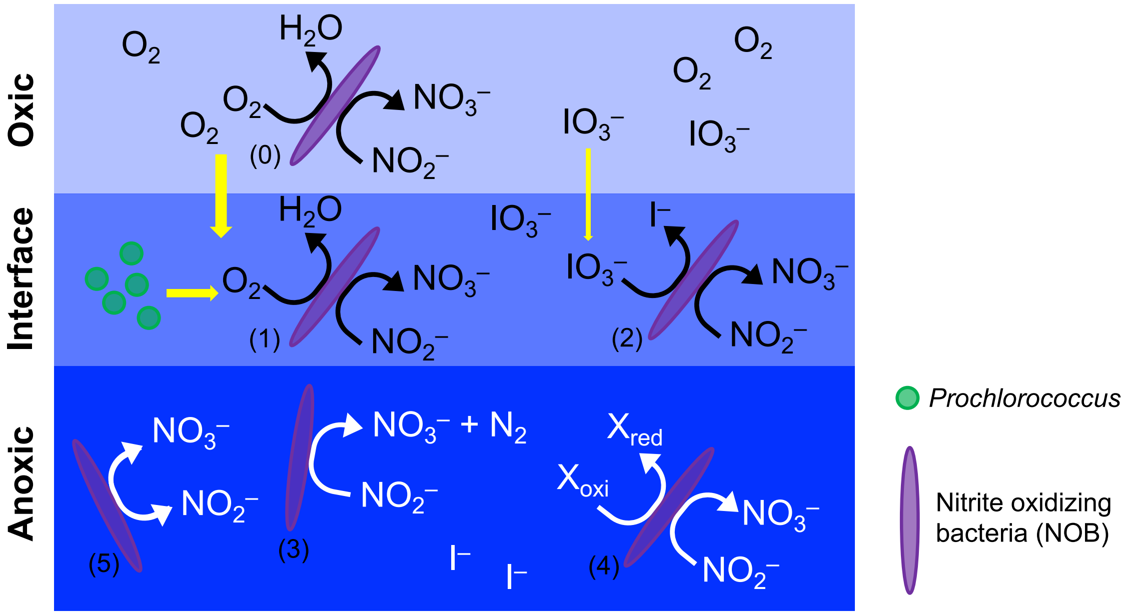

Figure S3. A proposed schematic of inferred niche differentiation of NOB along the redox gradient of the OMZ. In the oxic seawater, (0) NOB use oxygen to oxidize nitrite into nitrate. At the oxic-anoxic interface, (1) oxygen fluxes from the oxic layer and photosynthesis by *Prochlorococcus* at the deep chlorophyll maximum are sufficient to support nitrite oxidation by NOB. (2) Since oxygen concentration is below detection limit at the oxic-anoxic interface, iodate is thermodynamically possible as an alternative oxidant for nitrite oxidation. In the anoxic ODZ core, (3) nitrite oxidation occurs through disproportionation or (4) other undiscovered mechanisms independent of oxygen. (5) The bi-directional exchange between NO_2^- and NO_3^- catalyzed by nitrite oxidoreductase is also possible. X_{oxi} and X_{red} represent unknown oxidant and reduced form of that oxidant, respectively.

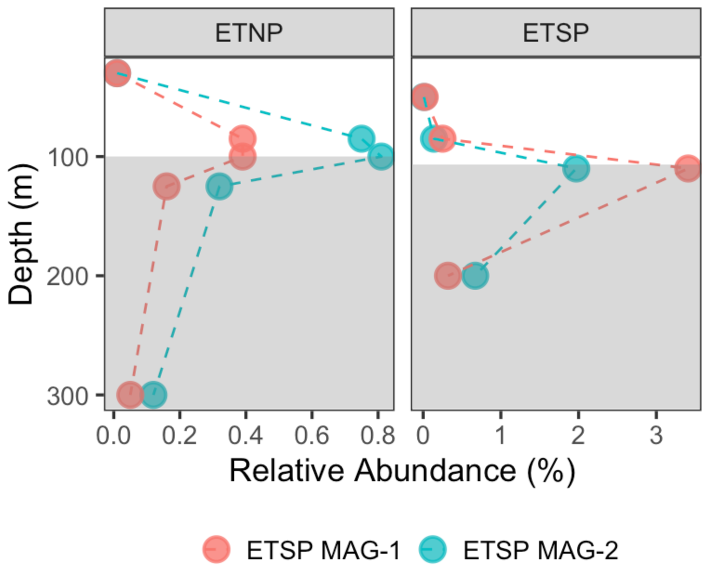

Figure S4. Transcriptional activity of two OMZ NOB 'species' in ETNP and ETSP OMZs. The transcriptional activity was estimated by mapping published metatranscriptomic reads from the ETNP OMZ (18°54'N, 104°54'W) [1] and the ETSP OMZ (20°07'S, 70°23'W) [2] to NOB MAG-1 and MAG-2 [3]. Shaded areas indicate the anoxic ODZ.

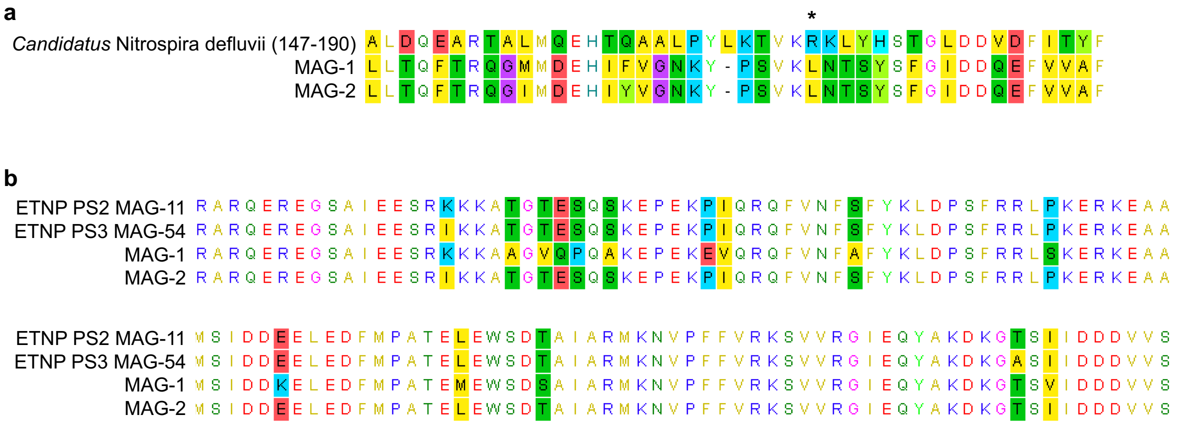

Figure S5. Alignment of Cld amino acid sequences. (a) the region of Cld from *Candidatus* Nitrospira defluvii which contains the arginine173 position [4] (indicated by a black star) and corresponding region of Cld from ETSP NOB MAGs [3], **(b)** the alignment of Cld from ETNP NOB MAGs (120 aa) and corresponding region of Cld from ETSP NOB MAGs.

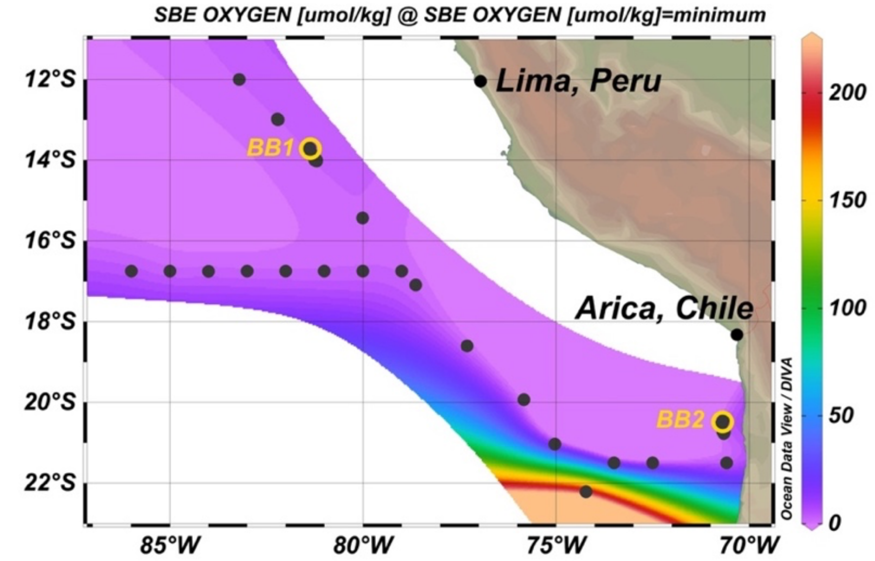

Figure S6. Locations of the two ETSP stations [5] **used in the inverse isotope model.**

Tables

Table S1 Sampling depths and accompanying information.

Station	Depth (m)	Feature	Seabird O_2 (μM)	Nitrite (μM)	Nitrate (μM)
PS2	90	oxycline	8.0	0.1	25
PS2	120	Top of ODZ	below detection limit	0.01	23
PS2	250	Core of ODZ	below detection limit	1.7	24
PS3	33	oxycline	2.2	0.1	24
PS3	50	oxycline	1.6	0.2	26
PS3	60	Top of ODZ	below detection limit	0.1	26
PS3	80	Top of ODZ	below detection limit	0.04	22
PS3	160	Core of ODZ	below detection limit	2.1	22

Table S2 Stoichiometries of nitrate producing processes.

Process	Stoichiometry
'Canonical' NO_2^- oxidation	$2\ NO_2^- + O_2 \rightarrow 2\ NO_3^-$
NO_2^- disproportionation[6, 7]	$5\ NO_2^- + 2\ H^+ \rightarrow N_2 + 3\ NO_3^- + H_2O$
Anammox[8]	$NH_4^+ + 1.146\ NO_2^- + 0.071\ HCO_3^- + 0.057\ H^+ \rightarrow$
	$0.986\ N_2 + 0.161\ NO_3^- + 2.002\ H_2O + 0.071\ CH_{1.74}O_{0.31}N_{0.20}$

Table S3 Average nucleotide identity (ANI) between two ETSP MAGs (MAG-1 and MAG-2) from Sun *et al.,* (2019) [3] and two ETNP MAGs identified in this study.

	MAG-1	MAG-2	ETNP PS2 MAG-11	ETNP PS3 MAG-54
**MAG-1**	100%	83.6%	83.8%	88.0%
**MAG-2**	83.6%	100%	99.5%	99.1%
**ETNP PS2 MAG-11**	83.8%	99.5%	100%	98.9%
**ETNP PS3 MAG-54**	88.0%	99.1%	98.9%	100%

Table S4 Genome characteristics of two *Nitrospina*-like MAGs obtained in this study and two from a previous study [3]**.**

	ETNP PS2 MAG-11	ETNP PS3 MAG-54	MAG-1 (Sun *et al.,* 2019)	MAG-2 (Sun *et al.,* 2019)
**Completeness**	37.9%	86.3%	93.1%	93.1%
**Contamination**	0.9%	46.3%	5.4%	3.9%
**Heterogeneity**	0.0%	20.7%	16.7%	0.0%
**MAG size (Mbp)**	0.7	2.9	2.2	2.2

Table S5 Relative abundance (RPKG) of the two MAGs from Sun *et al.,* (2019) [3] **in the core of the ODZ at ETNP stations PS2 and PS3.**

NOB species	Station	Relative Abundance (RPKG)
MAG-1	PS2	0.0007
MAG-2	PS2	0.0064
MAG-1	PS3	0.0059
MAG-2	PS3	0.0114

Table S6 Hypothesized reactions involved in NO_2^- disproportionation pathway.

Potential Enzyme	Reaction	
Nir	$2\ NO_2^- + 4\ H^+ + 2\ e^- \rightarrow 2\ NO + 2\ H_2O$	
Nxr	$3\ NO_2^- + 3\ H_2O \rightarrow 3\ NO_3^- + 6\ H^+ + 6\ e^-$	
Unknown Nod	$2\ NO \rightarrow N_2 + O_2$	a
Cytochrome *c* oxidase	$4\ H^+ + 4\ e^- + O_2 \rightarrow 2\ H_2O$	
	$5\ NO_2^- + 2\ H^+ \rightarrow N_2 + 3\ NO_3^- + H_2O$	b

[a] This reaction was potentially catalyzed by NO dismutase (Nod), predicted by Ettwig *et al*., (2010) [9].
[b] This is the overall equation predicted by van de Leemput *et al*., (2011) [7].

Table S7 Concentrations [5] **and isotopic** [10] **data used for modeling *in situ* nitrite oxidation rates at two stations (BB1 and BB2) in ETSP. *** Oxygen concentrations consistently around 2 μM are probably at analytical background since they are essentially at the Seabird sensor detection limit. The usage of STOX sensors [11] on the same cruise to confirm the low oxygen concentration (<10 nM) at these stations in the core of this ODZ was reported [12], and functional anoxia of the ODZ core was confirmed by STOX sensors at other stations in the ETSP and in the ETNP OMZ [13].

Station	Depth (m)	Nitrate (μM)	Nitrite (μM)	Ammonium (μM)	Oxygen* (μM)	$\delta^{15}_{Nitrate}$ (‰)	$\delta^{15}_{Nitrite}$ (‰)
BB1	301	27.7	4.5	0.16	2.4	15.9	-22.5
BB1	290	26.3	5.1	0.01	2.4	16.9	-21.5
BB1	251	21.0	7.2	0.05	2.4	22.4	-16.4
BB1	210	19.4	6.2	0.03	2.9	22.7	-14.7
BB1	199	18.6	6.3	0.03	2.3	23.6	-14.3
BB1	175	18.0	5.1	0.08	2.3	23.1	-14.3
BB1	151	14.5	6.0	0.05	2.2	25.8	-10.1
BB1	130	18.2	0.8	0.02	2.3	17.3	-15.8
BB2	375	29.8	1.7	0.01	2.6	13.7	-33.5
BB2	350	22.4	5.6	0.02	2.5	20.9	-20.6
BB2	325	19.2	6.7	0.01	2.4	24.6	-16.6
BB2	200	13.4	7.1	0.03	2.3	27.5	-10.2
BB2	150	10.9	7.1	0.03	2.3	30.9	-6.2
BB2	99	10.7	4.6	0.02	2.2	27.4	-5.4
BB2	95	10.6	4.6	0.03	2.2	27.9	-5.6
BB2	90	10.7	4.4	0.03	2.2	27.4	-5.6
BB2	86	11.1	4.0	0.03	2.2	26.6	-5.9
BB2	80	11.6	3.6	0.01	2.2	24.8	-4.8

References

1. Ganesh S, Bristow LA, Larsen M, Sarode N, Thamdrup B, Stewart FJ. Size-fraction partitioning of community gene transcription and nitrogen metabolism in a marine oxygen minimum zone. *ISME J* 2015; **9**: 2682–2696.
2. Stewart FJ, Ulloa O, DeLong EF. Microbial metatranscriptomics in a permanent marine oxygen minimum zone. *Environ Microbiol* 2012; **14**: 23–40.
3. Sun X, Kop LFM, Lau MCY, Frank J, Jayakumar A, Lücker S, et al. Uncultured Nitrospina-like species are major nitrite oxidizing bacteria in oxygen minimum zones. *ISME J* 2019; **13**: 2391–2402.
4. Kostan J, Sjöblom B, Maixner F, Mlynek G, Furtmüller PG, Obinger C, et al. Structural and functional characterisation of the chlorite dismutase from the nitrite-oxidizing bacterium ‘ Candidatus Nitrospira defluvii’: Identification of a catalytically important amino acid residue. *J Struct Biol* 2010; **172**: 331–342.
5. Sun X, Jayakumar A, Ward BB. Community Composition of Nitrous Oxide Consuming Bacteria in the Oxygen Minimum Zone of the Eastern Tropical South Pacific. *Front Microbiol* 2017; **8**: 1–11.
6. Strohm TO, Griffin B, Zumft WG, Schink B. Growth yields in bacterial denitrification and nitrate ammonification. *Appl Environ Microbiol* 2007; **73**: 1420–1424.
7. van de Leemput IA, Veraart AJ, Dakos V, De Klein JJM, Strous M, Scheffer M. Predicting microbial nitrogen pathways from basic principles. *Environ Microbiol* 2011; **13**: 1477–1487.

8. Oshiki M, Satoh H, Okabe S. Ecology and physiology of anaerobic ammonium oxidizing bacteria. *Environ Microbiol* 2016; **18**: 2784–2796.
9. Ettwig KF, Butler MK, Le Paslier D, Pelletier E, Mangenot S, Kuypers MMM, et al. Nitrite-driven anaerobic methane oxidation by oxygenic bacteria. *Nature* 2010; **464**: 543–548.
10. Peters BD, Babbin AR, Lettmann KA, Mordy CW, Ulloa O, Ward BB, et al. Vertical modeling of the nitrogen cycle in the eastern tropical South Pacific oxygen deficient zone using high-resolution concentration and isotope measurements. *Global Biogeochem Cycles* 2016; **30**: 1661–1681.
11. Revsbech NP, Larsen LH, Gundersen J, Dalsgaard T, Ulloa O, Thamdrup B. Determination of ultra-low oxygen concentrations in oxygen minimum zones by the STOX sensor. *Limnol Oceanogr Methods* 2009; **7**: 371–381.
12. Babbin AR, Peters BD, Mordy CW, Widner B, Casciotti KL, Ward BB. Multiple metabolisms constrain the anaerobic nitrite budget in the Eastern Tropical South Pacific. *Global Biogeochem Cycles* 2017; **31**: 258–271.
13. Tiano L, Garcia-Robledo E, Dalsgaard T, Devol AH, Ward BB, Ulloa O, et al. Oxygen distribution and aerobic respiration in the north and south eastern tropical Pacific oxygen minimum zones. *Deep Res Part I Oceanogr Res Pap* 2014; **94**: 173–183.
